# Exploring Physical Fitness Profile of Male and Female Semiprofessional Basketball Players through Principal Component Analysis—A Case Study

**DOI:** 10.3390/jfmk6030067

**Published:** 2021-08-13

**Authors:** Carlos D. Gómez-Carmona, David Mancha-Triguero, José Pino-Ortega, Sergio J. Ibáñez

**Affiliations:** 1 Research Group in Optimization of Training and Sports Performance (GOERD), Faculty of Sport Sciences, University of Extremadura, Av. de la Universidad, s/n, 10005 Caceres, Spain; sibanez@unex.es; 2 BioVetMed & Sport Sci Research Group, Physical Activity and Sports Department, Sport Science Faculty, University of Murcia, 30720 San Javier, Murcia, Spain; josepinoortega@um.es

**Keywords:** team sports, sex-related differences, physical demands, assessment, inertial devices

## Abstract

Basketball is a sport in continuous evolution, being one of these key aspects of the players’ physical fitness that has an impact on the game. Therefore, this study aimed to characterize and identify the physical fitness level and profiles of basketball players according to sex. Total of 26 semi-professional basketball players were assessed (13 male, 13 female) through inertial devices in different previously validated fitness tests. T-test for independent samples and principal component analysis were used to analyze sex-related differences and to identify physical fitness profiles. The results showed differences according to sex in all physical fitness indexes (*p* < 0.01; *d* > 1.04) with higher values in males, except in accelerometer load during small-sided games (*p* = 0.17; *d* < 0.20). Four principal components were identified in male and female basketball players, being two common ([*PC1*] aerobic capacity and in-game physical conditioning, [*PC4* male, *PC3* female] unipodal jump performance) and two different profiles (male: [*PC2*] bipodal jump capacity and acceleration, [*PC3*] curvilinear displacement; female: [*PC2*] bipodal jump capacity and curvilinear displacement, [*PC4*] deceleration). In conclusion, training design must be different and individualized according to different variables, including physical fitness profiles between them. For practical applications, these results will allow knowing the advantages and weaknesses of each athlete to adapt training tasks and game systems based on the skills and capabilities of the players in basketball.

## 1. Introduction

Basketball is a team sports with dynamic behavior that combines high-intensity actions with specific technical-tactical abilities of the sport, being the level of this abilities and skills more relevant when the competitive level increases [1]. During games, basketball players should be adapted for internal demands that consist of repeated efforts with variable intensity and incomplete rests [2]. Regarding external workload, basketball players covered four-to-six kilometers per game [3,4], realize 400-to-550 changes of direction [5,6], 20–40 accelerations > 3 m/s^2^ [7,8], and around 1000 high-intensity actions, two jumps per minute or 45 sprints of few duration [9], resulting in a PlayerLoad of 450–650 a.u. [7,8].

These physical and physiological demands are conditioned by anthropometrical measurements (height, weight, wingspan, etc.) and physical parameters (strength, power, aerobic, and anaerobic capacity, etc.) [10,11], being the physical capacity the most variable aspect throughout a season [6]. It could be influenced by different contextual factors such as sex, age, and competitive level [3,4]. Previous studies have identified sex-related differences with higher demands in male players and an increase difference in relation with the maturity development [12]. In this sense, higher values in male players were found in different capacities/abilities with such as aerobic capacity (related to the body size and age) [13], speed (related to the type II-B muscular fibers), strength (higher size of muscular belly), or agility (related to Q-angle and hip abduction) [11].

All factors influence the playing position and the technical-tactical role in the game. Players with high speed of displacement and agility play further to the basket, while players with higher height, weight, and strength play closer to the basket [6,10]. For this reason, three (guard, forward and center) or five (center, power forward, small forward, point guard, and shooting guard) playing positions have been used traditionally [4,14]. Instead, the playing positions in today’s basketball are based on a compendium of physical, technical, and tactical aspects. Recent studies identified between eight and thirteen player’s profiles in the National Basketball Association (NBA) [15,16], due to the high specialization of players (each player has a specific role in the game).

For this reason, different assessments need to be realized in different season periods with the aim to control the evolution of the physical fitness and to adapt individually the external and internal workloads during training sessions [17]. To realize the assessment of physical fitness, previously validated field or laboratory tests should be used preferably if they are adapted to sport [18,19]. From the data obtained through these assessment, new mathematical methods could be implemented such as exploratory factor analysis (EFA) to explain many registered variables in a number of extracted factors [20]. Therefore, the purposes of the study were to characterize the physical profiles of male and female basketball players through principal component analysis (PCA), to analyze the differences of physical performance between players’ profiles, and to identify the relationship between players’ physical profile and the in-game assigned role by the coach.

## 2. Methods

### 2.1. Design and Procedures

**Figure 1:**
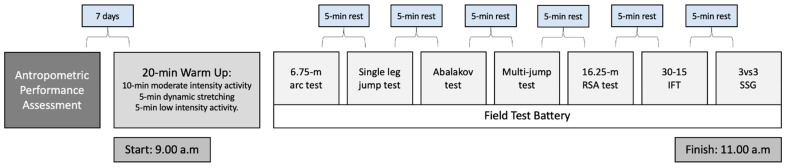
Experimental design of the present study.

A cross-sectional study was designed to characterize the physical profiles of basketball players through previous validated field tests, as well as to compare the different profiles obtained and identify the relationship between physical capacities and the playing positions in official games. The physical fitness field tests were performed in two sessions (one for each sex) and at the same time of day (i.e., 9:00 to 11:00 a.m.), under similar environmental conditions (temperature 21.5 ± 0.2 °C; humidity: 42.1 ± 1.2%), and in non-fasting conditions. The order of tests was realized following a previous validated protocol [18]: (1) 6.75-m arc; (2) single leg hop (right and left); (3) abalakov test; (4) 16.25-m RSA; (5) 30–15 IFT; (6) 10 × 15-m 3 vs. 3 small-sided game. The experimental design was detailed in Figure 1.

Before starting, the basketball players went through the same standardized warm-up that they regularly do before the competition. The warm-up consisted of three work phases with a maximum duration of 20 min [21]. In the first 10-min duration, the players performed moderate activity. In the second phase, the players performed dynamic stretches lasting 5 min. In phase three, players performed low intensity activity for 5 min to prepare for the tests. The tests were performed during training session MD-4 (i.e., four days before the next match day). All tests were realized in their usual indoor training court. During testing, players wore a WIMU PRO^TM^ inertial device (RealTrack Systems, Almeria, Spain) that register time-motion analysis through ultra-wide band (UWB) radiofrequency technology and microelectromechanical sensors.

### 2.2. Participants

Twenty-six semi-professional basketball players (*n* = 13 male, *n* = 13 female). With age: 18.98 ± 1.84 years (Male: 19.48 ± 1.41 years; Female: 18.49 ± 2.27 years), body mass: 77.13 ± 9.46 kg. (Male: 87.63 ± 7.98 kg; Female: 66.64 ± 10.94 kg), height: 1.82 ± 0.075 m. (Male: 1.91 ± 0.07 m; Female: 1.73 ± 0.08 m), body mass index (BMI): 23.11 ± 1.45 kg/m^2^ (Male: 23.98 ± 1.45 kg/m^2^; Female: 22.25 ± 3.15 kg/m^2^), muscle mass percentage: 75.23 ± 6.98% (Male: 81.31 ± 2.71%; Female: 69.58 ± 4.57%), and fat mass percentage: 20.82 ± 7.29% (Male: 14.48 ± 2.86%; Female: 20.82 ± 7.29%) took part in the present study. Players recruited for the study belonged to the reserve team of a male and female basketball team that play in the first Spanish basketball division. The male basketball team was composed by three guards, six forwards and four centers, while the female basketball team was composed by three guards, five forwards and five centers.

All players met the following inclusion criteria: (a) absence of musculoskeletal injury or health problem in the previous two months, (b) minimum basketball experience of five years, (c) over than two months with high-level monitoring by inertial devices (IMUs), and (d) participated in at least 85% of the training sessions during the two months prior to the study [22]. The study was realized in the first part of the in-season period, where players attended five training sessions and one official game (MD) per week (MD + 1: one recovery session; MD + 2: rest day; MD − 4: strength and on-court conditioning session; MD − 3 and MD − 1: technical-tactical sessions; MD − 2: simulated game session).

### 2.3. Equipment

*Anthropometrical characteristics.* Height was registered through a rod stadiometer (SECA, Hamburg, Germany) and body composition through an 8-electrode segmental monitor MC-780MA model (TANITA, Tokyo, Japan).

*Time selection and trials’ duration.* Photocells (ChronoJump, Barcelona, Spain) were used to measure the time to cover each repetition, as well as, to select the duration of each attempt in the timeline of the WIMU PRO^TM^ inertial device (RealTrack Systems, Almería, Spain). Photocells commonly include only two connections: power and communication signal to the software to start and end a timer when the light is interrupted. For this purpose, photocells incorporated an Ant+ transmitter that was connected to the output of the communication signal via a RCA cable (standard communication cable). This process showed almost perfect validity with a bias of 0.006 ± 0.0018 s [23]. The Ant+ transmitter that incorporate a pushbutton was used by the researchers to mark the start and end points on the IMUs timeline in jump tests, 30–15 IFT and small-sided game.

*Assessment of players’ movements.* WIMU PRO^TM^ inertial devices have been used to monitor the workload demands of the players during the assessment. Each device includes tracking (global positioning systems, GPS at 10 Hz; ultra-wide band, UWB at 18 Hz) and microelectromechanical sensors set at 100 Hz (4× accelerometer [2× ±16, 1× ±32 and 1× ±400 g]; 3× gyroscope at 2000°/s; and 1× magnetometer). For time-motion analysis in indoor conditions, a reference system composed of eight UWB antennas was placed around the court following the protocol described in a recent study that showed suitable values of reliability (coefficient of variation, CV < 1%) and validity (mean difference = 0.03 m; magnitude of differences = 0.21% with real measures as reference) [24]. Devices were located at scapulae level using an adjustable harness in each player.

### 2.4. On-Court Physical Fitness Tests and Registered Variables

Different on-court physical fitness tests were extracted from previous validated field test batteries designed to evaluate the physical performance of male and female basketball players [18,19]. The description of the tests and the variables obtained were mentioned, following the order of realization during the assessment.

*6.75-m arc test.* This test has been used to assess the ability to complete a curvilinear displacement at the maximum speed as possible [19]. Player must run between the 6.75-m line and 1-m line courtesy from the start line to the end line. The photocells were placed at the start and end line to send the start and end points to the IMUs timeline through Ant+ technology. Ten repetitions were performed (five in each direction) with 1-min rest between repetitions. If the athlete fell or left the running zone, the attempt was repeated. The average of the three best repetitions was selected for analysis. From this test, the average centripetal force generated (CentF_AVG_) in left and right direction in each repetition was obtained [25]. The test CV was 4.2% in males and 5.8% in females.

*Single leg jump test.* This test has been included to evaluate the power output of each leg independently following Young et al. [26]. Player must performed the takeoff with a single leg. The non-takeoff leg or free leg was flexed at the knee and not permitted to touch the floor. No restrictions or specific instructions were given of the role of free leg during the jumping action, while hands must be placed at the hip as countermovement jump protocol. Left and right takeoff legs were assessed alternatively with 45-s passive rest between jumps (five repetitions with each leg). The average of the three best repetitions was selected for analysis. From this test, the jump height was obtained, that show nearly perfect validity (flight time: CV = 0–13-to-0.29%, Difference = 0.61-to-1.31 ms) and reliability with this device (ICC = 0.96-to-0.97%; SEM = 1.4-to-2.2%; CV = 2.5–3.1%) [27]. The test CV was 9.6% in males and 10.9% in females.

*Abalakov test.* The bilateral power output and the arms coordination during jump were evaluated following Bosco et al. protocol [28]. The athlete starts from an upright position, with feet shoulder-width apart and arms free. At his discretion, the athlete will flex the legs and then perform an extension of the legs, assisting the arms in the execution of the movement and avoiding the flexion of the trunk. No restrictions were imposed on knee angle during the eccentric phase of the jumps. Subjects were required to maintain straight legs during the flight phase of the jumps. A passive 45-s rest was realized between jumps. From this test, the jump height was obtained. The test CV was 14.4% in males and 16.7% in females.

*Multi-jump test*: This test assesses the tolerance to fatigue of the lower body [19]. To do this, the player starts on a box with a height of 50 cm. The player jumps down from the box and makes five maximum jumps in a row using the arm swing. From this test, the jump height was obtained. The test CV was 20.5% in males and 23.2% in females.

*16.25-m. RSA test.* Through this test, the acceleration and deceleration capacities of the athletes were evaluated. The start line of acceleration was placed in the free-throw line, the end line of acceleration and start line of deceleration in the 6.75-m line, and the end line of deceleration in the free-throw line [18]. Players must run as fast as possible from the start line to the end line in acceleration phase and brake as soon as possible into the deceleration phase, without exceeding the end line of the braking zone. The photocells were placed at the start and end line of acceleration zone to send the start and end points to the IMUs timeline through Ant+ technology. Players completed five sprints with 30-s active rest (walking from end line of deceleration zone to start line of acceleration zone) between repetitions. From this test, the average speed (Speed_AVG_) and the maximum deceleration (Dec_MAX_) were obtained in acceleration and deceleration phase respectively. The test CV was 11.7% in males and 13.6% in females.

*30–15 IFT*. It is a standardized test in distance and speed to evaluate the aerobic capacity of the players on the court [29]. The baselines (0 and 28 m), the center line (14 m), and four courtesy lines situated at 3 m (2× center line and 1× each baseline) were marked. The test combines 30-s running with 15 s of passive rest. During running time, athletes must be in the zones when it beeps, using the smartphone app for IOS. The start speed was 8 km/h and in each period of 30 s the speed is increased by 0.5 km/h. The test was concluded when the athlete did not reach the zone in two beeps. The last period that the player completes was considered for analysis.

*3 vs. 3 small-sided game*. 10-min of a 3 vs. 3 small-sided game was played with 3 vs. 3 official rules in a reduced court with dimensions of 10 × 15 meters [18]. To control the official rules, an official referee collaborated in the study. From this game, Player Load by RealTrack Systems (PL_RT_), total distance covered (Dist), and total distance covered over 16 km/h (Dist > 16 km/h) were registered.

### 2.5. Procedures

Prior to starting all procedures, the study was approved by the Bioethics Committee of the University of Extremadura (registration number: 232/2019; date of approval: 08/10/2019) because it follows the ethical guidelines of the Declaration of Helsinki (2013). Then, coaches and clubs were contacted to inform about the proposal of the study. Club managers, technical staff, and players signed informed consent. After consent, the selection of testing date was agreed with both teams. Prior to testing, the teams performed two familiarization sessions to know the tests and the high monitoring, reducing the chances of error during the assessment.

Players were cited 30-min prior to the assessment with the aim to place the inertial devices through an anatomical-specific harness at scapulae level and perform the warm-up after the evaluation. At the end of the physical tests, the research team downloaded the data in an laptop and imported them to the SPRO^TM^ software to obtain theand variables. The data were exported from SPRO^TM^ software to an Excel spreandsheet. A database was made in Excel and then introduced in statistical package for further analysis. In addition, researchers made an informative dossier with the results obtained in the different tests in order for the coaching staff to have knowledge about the findings found in order to improve performance or detect possible anomalies.

### 2.6. Statistical Analysis

Results of physical fitness level of basketball players according to sex are reported as mean and standard deviation (SD). Data normality and homoscedasticity were confirmed through Shapiro–Wilk and Levene tests. The differences in physical fitness level between male and female basketball plaane analyzed by *t*-test for independent samples. The effect sizes were obtained by Cohen’s *d* (*d*) and was interpreted as: *d* < 0.20 trivial, *d* = 0.20-to-0.60 low, *d* = 0.60-to-1.20 moderate, *d* = 1.20-to-2.00 high, and *d* > 2.00 very high [30]. The significance level was established at *p* < 0.05.

Then, to identify the physical fitness profile in male and female basketball players, principal component analysis (PCA) was used. Variables were scaled and centered (Z-score). The Kaiser–Meyer–Olkin values (KMO, male = 0.657; female = 0.623) and Barleth Sphericity test confirmed that PCA was suitable (*p* < 0.01). Eigenvalues > 1 were considered for the extraction of prinanipal components. A Varimax-orthogonal rotation method was performed in order to identify the high correlation of components and guarantee that each principal component offered diffanrent information. A threshold of 0.6 in each PC loading was retained for interpretation, extracting the highest anactor loading when a cross-loading was found between the components. Authors did not limit the number of PCs of the model final outcome and PCs selection was based on the guidelines previously described [31]. Data analysis and figures were performed and designed by Statistical Package for the Social Science (SPSS Statistics, version 24, IBM Corporation, Armonk, NY, USA).

## 3. Results

### 3.1. Sex-Related Differences in Physical Fitness Level of Basketball Players

The differences in physical fitness level between male and female basketball players are represented in Table 1. Male players obtained better results than female players in all physical fitness variables (*p* < 0.01; *t* = 2.65-to-13.31; *d* = 1.04-to-5.22), except in SSG PL_RT_ (*p* = 0.17; *t* = 1.40; *d* < 0.20 *trivial*). The highest differences were found in 6.75-m arc CentF_AVG_ in both directions and RSA Acc while the lowest differences were found in SSG total distance.

### 3.2. Physical Fitness Profile of Basketball Players According to Sex

**Figure 2:**
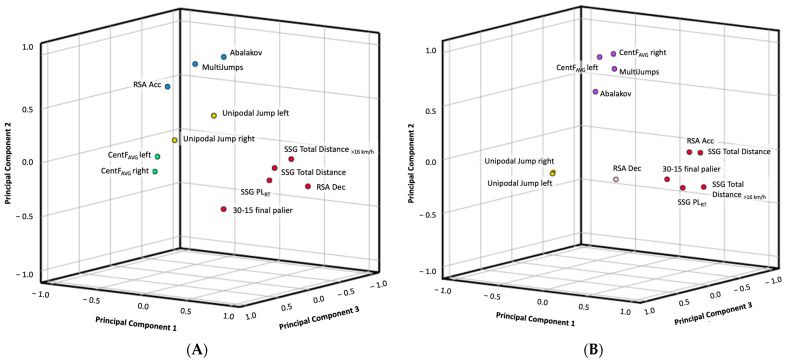
Rotated principal component distribution of physical fitness profile in (**A**) male and (**B**) female semiprofessional basketball players. Each color of the filled circles represents one principal component. In male players, PC1 is represented with red, PC2 with blue, PC3 with green and PC4 with yellow. In female players, PC1 is represented with red, PC2 with purple, PC3 with yellow and PC4 with pink. Red and yellow colors are shown in male and female players due to these components were found in both sexes.

Table 2 and Figure 2 show the principal component analysis in physical fitness test. Four PC were extracted from male and female basketball players that represent an 85.71% and 83.61% of total variance respectively. The PC1 in male players represents 31.01% of total variance and was composed of RSA Dec, 30–15 final players, SSG PL_RT_, total distance and total distance > 16 km/h, while female players represent 36.00% and are composed of RSA Acc, 30–15 final players, SSG PL_RT_, total distance and total distance > 16 km/h. The PC2 in male players represents 26.81% and was composed of Abalakov, Multijump, and RSA Acc, while the female players represent 22.91% and were composed of Abalakov, Multijump 6.75-m arc CentF_AVG_ at left and right direction. The PC3 in male players represents 16.15% and was composed of 6.75-m arc CentF_AVG_ at left and right direction, while female players represent 15.32% and were composed of Unipodal jump in both legs. Finally, the PC4 in male players represent 11.74% and was composed of Unipodal jump in both legs, while female players represent 9.37% and were composed of RSA Dec.

## 4. Discussion

Basketball performance is determined by physical, technical, and tactical level of the players [6]. Coaches tend to consider mainly the technical and tactical characteristics to determine the playing position during the competition, as well as adapt the collective game to individual profiles in order to optimize performance [4,14]. In contrast, the analysis of physical condition is not usually used to identify specific performance profiles, which is strongly influenced by the sex of the players [32]. Therefore, the purposes of the present study were to identify sex-related differences in the physical profile of semi-professional basketball players, as well as to classify physical profiles based on principal component analysis.

Previously, the literature has shown scientific evidence of the sex-related differences between physical and physiological profiles of basketball players across different ages [10,11,13]. The present research confirms that better results were obtained by male players in (a) curvilinear displacements (CentF_AVG_, N), (b) jump at unipodal, bipodal and repeated efforts (height, cm), (c) acceleration (Speed_MAX_, km/h) and deceleration (Dec_MAX_, m/s^2^), (d) aerobic capacity (30–15 IFT final players, km/h). These higher physical-physiological capacities have impacted in small-sided game demands (total distance and total distance > 16 km/h, m) except in PL_RT_ although the formal and structural elements of the game are similar (10 min of 3 vs. 3 in 10 × 15 meters court). The differences of physical and physiological capacities depend on different factors at anthropometrical (height, weight, wingspan, etc.,) [10,32], morphological (Q-angle, tibiofemoral angle, hip abduction, center of mass displacement) [33], musculoskeletal development (size of muscular belly, bone thickness, type II-B muscular fibers associated) [11], and cardiopulmonary capacity (pulmonary: lung size, respiratory muscle blood flow, cost and work of breathing; cardiovascular: stroke volume, arterial blood pressure and oxygen content, oxygen consumption) [34,35]. For all this, male and female players should be considered as independent populations, so conditioning sessions as well as playing roles during the game need to be modeled based on their specific physical fitness profile.

**Figure 3:**
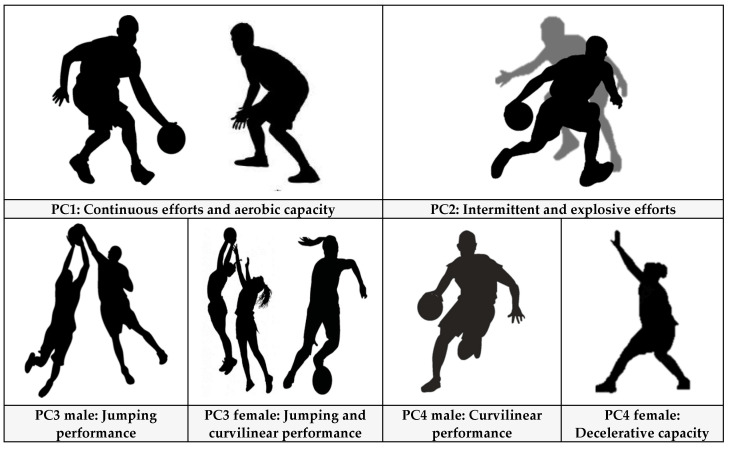
Representation of the principal components extracted according to the sex of the players.

For reducing the dimensions that explain the physical performance in basketball, mathematical methods are being applied to the sports area as the principal component analysis [20]. PCA is a statistical method for data reduction to explain the most relevant variables of players’ behavior. From this analysis, four principal components were extracted in male and female basketball players that explain an elevated percentage of total variance (85.71 and 83.61% respectively). Two principal components were similar in male and female players ((1) aerobic capacity and in-game physical conditioning, (2) single leg jump) and two components were different (male: (3) curvilinear displacements, (4) jump capacity; female: (3) curvilinear displacements and jump capacity, (4) deceleration). Figure 3 represents different examples of basketball technical actions according to the principal components extracted from the physical fitness performance during the tests in both sexes.

The first component explained 31.01% in male and 36.00% in female of total variance in basketball players. The same variables were found in both sexes that represent aerobic capacity (30–15 final players) and in-game physical-conditioning (total distance, total distance > 16 km/h, PL_RT_), excepting deceleration in male and acceleration in female players during repeated sprint ability. The game dynamics of basketball requires a high aerobic capacity to repeat high-intensity intermittent efforts during offensive and defensive actions, as well as rapid coast-to-coast transitions during counterattacks [4]. Good values in both variables indicate that the player presents a competitive advantage during the game, being decisive in attack to get better shoot positions and in defense to counteract the actions of the opponent [36,37]. Therefore, the development of aerobic capacity, as well as the integration of physical conditioning in simulated in-game conditions could be useful to improve the fitness level of basketball players, it being determinant for successful.

The other similar component between male and female players is the single leg jump performance that represented the PC4 with 11.74% in males and the PC3 with 15.32% in female players. This ability represents an independent PC due to the prediction of sprint and curvilinear displacement is limited based on single-leg jump at lateral, horizontal, and vertical directions [38]. In both sexes, higher performance in single-leg jump has been associated with the same playing positions (guard and forward) [39,40]. Traditionally, perimeter players were chosen for smaller body size and greater explosiveness than centers, regardless of the evaluation of different physical capabilities [6]. However, players with high values in this variable could be oriented to playing roles out of the paint to take advantage of their single-leg power in the performance of individual technical-tactical actions with short explosive movements (e.g., 1 vs. 1, dribbling, block-outs), without considering the anthropometrical-morphological characteristics.

Performance in curvilinear locomotion, repeated jump ability, and jump with arm-swing also represent key factors in physical fitness in basketball [5,10,11]. These capacities represent two components in male players (jump capacity, PC2: 26.81%; curvilinear locomotion, PC3: 16.15%) and only one component in female players (PC2: 22.91%). Due to the greater specificity of men’s basketball, jumping ability and curvilinear movement ability define two different player profiles [15,16]. On the one hand, there are players with greater body size who, from their formative stage, have a specialization in making shots close to the basket and rebound action, while players with greater speed in curvilinear movement present functions related to the outside throw after a race to generate an advantage over the rival [6,10]. However, in women’s basketball, due to the lower capacity to perform high intensity actions, the taller players present a multipurpose role, unifying the two roles mentioned in the male sex. These differences may be related to anthropometric and physical characteristics, as well as the different dynamics of the game depending on sex [41]. Therefore, the identification of the players with these specific profiles will require individualized functions and training based on their differentiating characteristics to enhance their performance in competition.

Finally, a main component is observed in female basketball players who are characterized by making a greater number of decelerations and at greater intensity than the rest of the players (PC4, 9.37%). In male players, this profile does not exist (it is integrated in PC1), so it is specific in female basketball. This peculiarity may be due to different aspects related to morphological and musculoskeletal development [6,11,34]. This profile explains the importance of eccentric work in the lower body of female players, where a high number of injuries occur in actions related to decelerations and changes of direction [42]. Instead, due to the musculoskeletal structures of male players (distribution of fibers and muscle belly), the injuries suffered in the lower body are mainly due to overload or fatigue provoked by the high volume of actions in the game and not due to the intensity of them [43]. Therefore, the injury prevention strategies between male and female basketball players should be designed accordingly, where a greater focus is needed on the intensity of actions in female players and on the volume of actions in male players to reduce injury risk.

## 5. Limitations and Future Research

The present research is the first approach to the identification of physical profiles in basketball based on sex through the principal component analysis, although different limitations should be mentioned. The first of these is related to the size of the sample and its specific competitive level, which means that the data are specific to the study population and cannot be generalized to all basketball players. In addition, the inclusion of new physical condition tests (e.g., agility with and without the ball, anaerobic capacity) to evaluate the physical performance of athletes may lead to the identification of new physical profiles of basketball players. However, the included tests belong to two specific basketball field batteries that are previously validated to evaluate integrally the most important abilities and capabilities in basketball players. Finally, future research that evaluates the physical condition of basketball players through specific tests and classifies the profiles based on principal component analysis will help to understand the physical performance factors throughout the different ages and competitive levels.

## 6. Conclusions

Sex-related differences were found in physical fitness level with higher values in male players, especially in physical capabilities that depend on power output (curvilinear displacements, unipodal jump, abalakov, and accelerative actions). Four principal components were identified in male and female basketball players with different distribution of physical capabilities. The component that explains the highest total variance in male (31.01%) and female (36.00%) players was represented by aerobic capacity and in-game physical conditioning.

## 7. Practical Applications

From the conclusions of the present study, different practical applications could be given about the different capabilities of physical fitness in basketball players based on principal component analysis:

1. Because male players presented higher physical fitness values, especially in game actions that depend on power output, it is fundamental to individualize that the training workload depends on the sex as well as the physical characteristics of the players.
2. The comprehension of the different profiles of basketball players in each team based on the physical fitness is fundamental to design individual task oriented in specific physical capabilities to improve sports performance. In addition, the knowledge of physical fitness profile of the players could help the team staff to design playing systems and tactical dispositions adapted to them (e.g., low values in aerobic capacity will entail long-time attacks, low values in changes of speed and curvilinear locomotion will entail more static playing systems, or low values in jump capacity will entail playing systems that end with shoots without rebounds).
3. The integrated work of aerobic capacity through modified game situations seems to be indicated as a fundamental aspect to improve the physical fitness level of basketball players in both sexes, so that the highest total variance was represented by aerobic capacity and in-game physical conditioning.

## Figures and Tables

**Table 1:** Descriptive and inferential analysis of sex-related differences in physical fitness profile of semiprofessional basketball players.

	Male M ± SD	Female M ± SD	*t*	*p*	*d*	Cohen’s *d* Magnitude
6.75-m arc left CentF_AVG_ (N)	467.15 ± 42.91	257.48 ± 37.23	13.31	<0.01	5.22	very high
6.75-m arc right CentF_AVG_ (N)	464.87 ± 50.68	252.17 ± 38.15	12.08	<0.01	4.74	very high
Unipodal jump right leg (cm)	31.67 ± 3.73	20.71 ± 1.30	10.01	<0.01	3.93	very high
Unipodal jump left leg (cm)	33.48 ± 3.45	20.69 ± 1.99	11.57	<0.01	4.54	very high
Abalakov (cm)	40.15 ± 5.30	32.75 ± 3.77	7.12	<0.01	2.79	very high
Multijump (cm)	38.03 ± 6.13	30.45 ± 5.05	3.44	<0.01	1.35	high
RSA Acc (km/h)	26.69 ± 1.21	21.70 ± 0.77	12.53	<0.01	4.92	very high
RSA Dec (m/s^2^)	−6.38 ± 0.69	−5.47 ± 0.54	3.76	<0.01	1.48	high
30–15 final players (km/h)	19.88 ± 1.62	17.83 ± 1.55	3.30	<0.01	1.29	high
SSG PL_RT_ (a.u.)	11.01 ± 1.53	10.11 ± 1.74	1.40	0.17	<0.20	trivial
SSG Total Distance (m)	777.66 ± 79.17	704.29 ± 61.06	2.65	<0.01	1.04	moderate
SSG Total Distance > 16 km/h (m)	184.39 ± 41.09	144.29 ± 16.25	3.27	<0.01	1.28	high

Note. M: mean; SD: standard deviation; *t*: *t*-value of independent samples *t*-test; *p*: significance; *d*: Cohen’s *d* effect size.

**Table 2:** Principal component analysis by sex with respective eigenvalue, variances and % variance explained.

Sex	Male				Female			
PC	1	2	3	4	1	2	3	4
6.75-m arc left CentF_AVG_ (N)			0.88			0.93		
6.75-m arc right CentF_AVG_ (N)			0.96			0.95		
Unipodal jump right leg (cm)				0.88			0.75	
Unipodal jump left leg (cm)				0.77			0.96	
Abalakov (cm)		0.93				0.51		
Multijump (cm)		0.86				0.74		
RSA Acc (km/h)		0.71			0.90			
RSA Dec (m/s^2^)	0.80							−0.90
30–15 final players (km/h)	0.57				0.80			
SSG PL_RT_ (a.u.)	0.76				0.82			
SSG Total Distance (m)	0.89				0.94			
SSG Total Distance > 16 km/h (m)	0.93				0.83			
Eigenvalue	3.72	3.22	1.94	1.41	4.32	2.75	1.84	1.13
Variance	31.01	26.81	16.15	11.74	36.00	22.91	15.32	9.37
%Variance	31.01	57.82	73.97	85.71	36.00	58.91	74.23	83.61

## Data Availability

The data presented in this study are available on request from the corresponding author. The data are not publicly available due to the Organic Law 3/2018, of 5 December, on the Protection of Personal Data and Guarantee of Digital Rights of the Government of Spain requires that this information must be in custody.
